# Editorial: The acute and long-term impact of COVID-19 on mental health of children and adolescents

**DOI:** 10.3389/fpsyt.2023.1265934

**Published:** 2023-08-17

**Authors:** Kai Yuan, Yanping Bao, Yue Leng, Xiaoyu Li

**Affiliations:** ^1^Peking University Sixth Hospital, Peking University Institute of Mental Health, NHC Key Laboratory of Mental Health (Peking University), National Clinical Research Center for Mental Disorders (Peking University Sixth Hospital), Chinese Academy of Medical Sciences Research Unit (No. 2018RU006), Peking University, Beijing, China; ^2^National Institute on Drug Dependence, Peking University, Beijing, China; ^3^School of Public Health, Peking University, Beijing, China; ^4^Department of Psychiatry and Behavioral Sciences, University of California, San Francisco, San Francisco, CA, United States; ^5^Department of Sociology, School of Social Sciences, Tsinghua University, Beijing, China

**Keywords:** children, adolescents, COVID-19, mental health, long COVID, depression

The COVID-19 epidemic has dramatically paralyzed the world, which has also caused mental health problems among the public, especially vulnerable groups like children and adolescents. As a traumatic event, the outbreak of COVID-19 might increase the prevalence of a series of mental health problems in children and adolescents probably due to social distancing, school closures, as well as separation from parents and home quarantine (1). It is also noticed that COVID-19 patients may experience long-term post-acute COVID-19 symptoms, or long COVID, which describes a wide range of persistent neurological and neuropsychiatric symptoms developed during or following an infection, including cognitive impairments, depression, and anxiety (2).

In this regard, analyzing the impact of COVID-19 on mental health of children and adolescents from both the acute and long-term perspective is of great significance (3). On the one hand, children and youth are often politically neglected because they seem less likely to be infected early in the pandemic, leaving a less important burden of acute COVID-19; On the other hand, children and adolescents are in critical periods of neurodevelopment and once they develop mental health problems, the long-lasting negative influence might span across their lifetime.

The goal of this edition of Research Topic is mainly to highlight the mental health problem, substance use disorders, deviant behaviors and cognition impairment of children and adolescents during the COVID-19 pandemic, as well as related risk factors. Furthermore, we also aim to illustrate the persistent neurological, neuropsychiatric, and neurodevelopmental complication or sequelae among the pediatric COVID-19 survivors. Finally, we want to investigate effective and affordable interventions, especially online interventions, of mental health disorders of children and adolescents related to the COVID-19 outbreak.

These aims were addressed by a total of 18 studies, 3 of which reviewed life change and mental health problems caused by COVID-19 pandemic in children and adolescents. Guo et al. analyzed 5,594 relevant literatures, identifying mental health and life change as prominent themes. The findings provide valuable insights into trends and gaps, serving as a reference for future research, decision-making, and systematic reviews. Additionally, the study by Han et al. highlights the increased risk of gaming disorder during social isolation, emphasizing the need for mental health providers to educate children, adolescents, and parents about healthy stress relief alternatives and effective parental control measures.

Ng and Ng revealed that children from low socioeconomic status families, with parents having low educational attainment and living in small homes, are more vulnerable to mental health problems. Protective factors, including parents' resilience, positive parent-child relationships, and school connectedness, should be emphasized. By understanding and applying the findings and recommendations from these studies, policymakers, healthcare providers, educators, and parents can collaborate to develop effective interventions and support mechanisms to safeguard the mental wellbeing of children and adolescents during and beyond the COVID-19 pandemic.

Several research articles in our topic have shed light on the mental and psychological challenges encountered by children and adolescents. Shi et al. conducted a web-based survey and discovered that anxiety and sleep disturbances significantly improved after quarantine, while depression showed no alleviation. They also found bidirectional relationships between anxiety, depression, and sleep disturbance, with depression and sleep disturbances predicting post-quarantine mental health issues. In a cross-sectional online survey study, Nin et al. identified two clusters with varying stress levels, linked to differences in personality traits, quality of life, depression, and anxiety. Neuroticism was identified as a risk factor, while extraversion acted as a protective factor against pandemic-related stress. This study introduced a taxonomy of factors that influence stress sensitivity, providing indicators of psychological distress and quality of life during the pandemic.

Apart from cross-sectional study, longitudinal research by Martinsone et al. examined the impact of the pandemic on adolescents' mental health over time. They found that changes in social emotional skills were associated with changes in mental health outcomes, emphasizing the importance of addressing social emotional learning to enhance resilience and wellbeing. Additionally, Maouch et al. investigated the effects of disrupted activity systems on mental wellbeing. They discovered significant associations between activity system components and PTSD, reflective function, and schizotypal traits. The study highlighted how the disturbance of life narratives during the pandemic can significantly impact mental health. Collectively, these studies provide valuable insights into the mental and psychological problems encountered by children and adolescents.

Several studies have focused exclusively on depression symptoms precipitated by the pandemic in children and adolescents. A recent study has emphasized the perturbing rates of depression and its relationship with self-efficacy in Iranian children during the COVID-19 outbreak (Zakeri et al.). The research involved 321 children and utilized the Children's Depression Inventory (CDI) and Self-Efficacy Questionnaire for Children (SEQ-C). Interestingly, while no overall significant correlation was found between depression and self-efficacy, subscales of the CDI including negative mood, ineffectiveness, and negative self-esteem had a significant relationship with self-efficacy. Furthermore, family income, risk of coronavirus infection, effectiveness of preventive measures, and the source of information about the coronavirus disease were found to have a substantial association with depression rates.

Another study conducted among university students in Hong Kong highlighted the need for further investigation into the determinants and mitigating factors of depression within this demographic. The study, which utilized the Center for Epidemiologic Studies Depression Scale Revised (CESD-R) to assess depressive symptoms in 1,648 students, found a substantial 48.4% prevalence of “at-risk” scores for clinical depression (Shek et al.). Socio-demographic factors including age, gender, student status, and financial hardship were identified as significant correlates of depressive symptoms. Additionally, the study investigated the role of need satisfaction and positive youth development (PYD) attributes, such as resilience, emotional competence, and family functioning, in predicting depression rates. These factors were found to negatively predict depression, with all PYD attributes demonstrating a moderating effect on the predictive impact of need satisfaction on depression. The results above underline the importance of comprehensive intervention strategies and research efforts to mitigate and prevent depression among children, particularly in the context of global health crises.

It is noteworthy that the psychological distress of this era has been exacerbated by certain aspects of media engagement. A compelling investigation into media's influence on PTSD symptoms among college students revealed that the media content itself, rather than the source, significantly impacted the students' mental health (Zhu et al.). Positive media content correlated negatively with PTSD symptoms, emphasizing the role of constructive narratives in mental health resilience. Concurrently, students afflicted with PTSD symptoms displayed a reduced willingness to engage in efficient online learning, hinting at potential educational setbacks caused by the intersection of media exposure, information overload, and mental health disorders.

A natural experiment in Switzerland further expounded on this by illuminating the long-term consequences of increased screen time on adolescents' mental health during the lockdown (Marciano et al.). The study found that most mental health problems, including anxiety, depression, and inattention, escalated over time with a medium effect size. Interestingly, an increase in time spent on social media was significantly associated with deteriorating mental health, while more structured media activities, such as television viewing, was likely to mitigate levels of inattention and anxiety. These studies underscore the intricate and nuanced impact of media exposure on young minds, thereby prompting a pressing need for further studies on effective media practices during public health crises to safeguard the mental health of future generations.

Building upon the exploration of media exposure's effects on children and adolescents' mental health, it becomes imperative to delve into the grave subject of suicide among this population during the COVID-19 pandemic. A nationwide survey in South Korea, utilizing nomogram techniques with a sample size of 54,948 adolescents, developed a model to identify cohorts vulnerable to suicidal ideation in the pandemic's aftermath (Byeon). The findings identified 8th graders with recent experiences of depression, heightened subjective stress, loneliness, decreased household economic status, and poor academic performance as particularly susceptible to suicidal ideation amid the COVID-19 crisis. The impressive precision of the prediction model, developed using logistic regression and Extreme Gradient Boosting (XGBoost), accentuates the urgency to acknowledge the severity of adolescent suicide and mental health in the wake of the pandemic. It further emphasizes the necessity for implementing customized support systems at community and school levels.

Another investigation resonated with these findings, demonstrating an escalation in the incidence of Slovene youth necessitating urgent psychiatric intervention due to suicidal ideation and attempts, specifically during the period of the COVID-19 pandemic (Kirič et al.). Upon analysis, the increase did not seem to align directly with periods of school closure, but instead exhibited a stronger correlation with the overall duration of the pandemic. The study stressed the importance for medical personnel to readily interpret these probabilities, thereby enabling the identification of children and adolescents at heightened risk. The results reiterate the paramount importance of a comprehensive support system, specifically advocating for characteristics that accommodate the unique needs of those individuals at an elevated risk of suicide amid the health crisis.

Finally, in the context of COVID-19, the critical role of anxiety as an intermediary between impulsivity and suicidal ideation among patients with major depressive disorder (MDD) is examined. Given the common co-occurrence of anxiety symptoms with depression, the exploration of its impact on suicidal ideation from the perspective of impulsivity becomes a priority. Findings from a study revealed that major depressive disorder (MDD) patients with high suicidal ideation exhibited more trait anxiety, albeit no discernible differences were found in state anxiety and impulsivity when compared to those with low suicidal ideation (Cheng et al.). Intriguingly, trait anxiety was found to mediate fully between impulsivity and suicidal ideation. This emphasizes the need for heightened attention toward MDD patients with anxiety symptoms, serving as a potent prevention and intervention measure against suicidality.

The COVID-19 pandemic has also caused long-term psychological effects on the youth. The study by Liao et al. provides evidence of the significant prolonged psychological burden among Chinese undergraduate medical students caused by the pandemic. They conducted a longitudinal survey, tracking the mental burden changes among 863 Chinese undergraduate medical students before, during, and after the outbreak. The findings revealed an increase in the prevalence of overall mental burden from 27.46 to 37.28% after the COVID-19 outbreak. Specifically, the prevalence of stress reaction symptoms decreased (from 10.90 to 3.60%), while the rates of psychological distress (from 28.06 to 37.95%) and insomnia symptoms (from 12.54 to 20.71%) increased. The study also identified several risk factors, such as obsessive-compulsive symptoms, somatic symptoms, internet addiction, childhood adversity, stressful life events, and neuroticism, which were associated with a higher risk of developing mental health problems. Conversely, healthy family function and extraversion were found to have a positive impact on mental burden.

Similarly, the study by Wei et al. highlights the ongoing impact on the mental health of first-year college students who encountered more challenges and suffer more mental health problems in the post-COVID-19 era. The research, based on the study demands-resources (SD-R) model, examined the effects of time pressure, perceived social support, emotional exhaustion, and student engagement on mental health. The findings revealed that time pressure and perceived social support were key factors influencing the mental health of first-year college students in the post-COVID-19 era. These findings emphasize the need for continued mental health care for students, even after the COVID-19 outbreak, particularly for those exhibiting persistent or worsening symptoms.

In the face of increasing psychological issues among children and adolescents especially the post-acute COVID-19 syndrome, it is crucial to develop effective strategies to help them overcome these challenges. A study conducted by Wu et al. examined the relationship between self-efficacy, self-disclosure, and coping strategies among 585 patients with moderate to severe depression, aged 11 to 24. The findings suggested that encouraging self-disclosure and sharing emotions and concerns significantly reduced their psychological stress. Additionally, fostering their self-efficacy, belief in their ability to cope with difficulties and challenges, facilitated their selection of proactive coping strategies. Furthermore, providing online physical activity programs, such as High-Intensity Interval Training (HIIT), proved to be an effective intervention in improving mental health and emotional wellbeing, as discussed in Philippot et al.'s study on the impact of an online HIIT program on clinical psychological symptoms among higher education students. The study revealed significant improvements in clinical stress and depressive symptoms among students who participated in a four-week online HIIT program during the COVID-19 pandemic. Therefore, by integrating these strategies, we can better assist children and adolescents in dealing with psychological issues and promoting their healthy neurodevelopment.

In conclusion, the COVID-19 pandemic has had both acute and long-term psychological impact on children and adolescents. They have experienced increased loneliness, anxiety, and depression due to prolonged social isolation and restrictions. Additionally, the disruption to their education and development may lead to learning difficulties and future mental health problems. Though the COVID-19 pandemic has presented unprecedented challenges, it has also opened up an opportunity to prioritize and enhance mental health support for young individuals. We hope our Research Topic could raise attention of the public on mental health problems of children and adolescents caused by the pandemic and provide continuous support and care to help them navigate through these challenging times.

## Author contributions

KY: Conceptualization, Writing—original draft. YB: Writing—review and editing. YL: Conceptualization, Writing—review and editing. XL: Conceptualization, Writing—review and editing.
